# Different IgE recognition of mite allergen components in asthmatic and nonasthmatic children

**DOI:** 10.1016/j.jaci.2015.03.024

**Published:** 2015-10

**Authors:** Yvonne Resch, Sven Michel, Michael Kabesch, Christian Lupinek, Rudolf Valenta, Susanne Vrtala

**Affiliations:** a Division of Immunopathology, Department of Pathophysiology and Allergy Research, Center for Pathophysiology, Infectiology and Immunology, Medical University of Vienna, Vienna, Austria; b Department of Pediatric Pneumology and Allergy, University Children's Hospital Regensburg (KUNO), Regensburg, Germany; c Christian Doppler Laboratory for the Development of Allergen Chips, Medical University of Vienna, Vienna, Austria

**Keywords:** House dust mite allergy, asthma, allergen microarray, recombinant allergens, IgE, IgG, diagnosis, HDM, House dust mite, ISAC, Immuno Solid-phase Allergen Chip, ISU, ISAC standardized units, OR, Odds ratio

## Abstract

**Background:**

House dust mites (HDMs) represent one of the most important inducers of respiratory allergies worldwide.

**Objective:**

We sought to investigate the IgE and IgG reactivity profiles to a comprehensive panel of HDM allergens in children with allergic asthma and to compare them with those of nonasthmatic atopic children.

**Methods:**

Sera from clinically well-characterized asthmatic children with HDM allergy (n = 105), nonasthmatic children (n = 53), and nonatopic nonasthmatic children (n = 53) were analyzed for IgE and IgG reactivity to a panel of 7 HDM allergens (nDer p 1, rDer p 2, rDer p 5, rDer p 7, rDer p 10, rDer p 21, and rDer p 23) by means of allergen microarray technology.

**Results:**

Asthmatic children with HDM allergy more frequently showed an IgE response to each of the HDM allergens and recognized more allergens than nonasthmatic children with HDM allergy. Furthermore, IgE levels to certain HDM allergens (nDer p 1, *P* = .002; rDer p 2, *P* = .007; rDer p 5, *P* = .031; and rDer p 23, *P* < .001) were significantly higher in asthmatic children than in children without asthma. By contrast, fewer asthmatic children showed IgG reactivity to HDM allergens than nonasthmatic children, but allergen-specific IgG levels were comparable.

**Conclusion:**

The IgE and IgG reactivity profiles to HDM allergens, as well as IgE levels to certain allergen components, differed considerably between children with and without asthmatic symptoms caused by HDM allergy. In fact, asthmatic children were characterized by an expanded IgE repertoire regarding the numbers of recognized allergen components and by increased specific IgE levels.

Exposure and atopic sensitization to indoor allergens, such as those from pets, molds, cockroaches, and house dust mites (HDMs), are known to be strongly associated with the development of asthma.^1-3^ The association between asthma and house dust was reported in 1921 by Kern,^4^ who showed that many asthmatic patients had strongly positive skin test responses to house dust. Since 1967, when mites of the genus *Dermatophagoides* were determined to be a major allergen-producing source in house dust,^5^ many studies have confirmed the importance of HDM allergy as a major risk factor for asthma.^6-8^ In fact, up to 85% of asthmatic patients are allergic to *Dermatophagoides pteronyssinus*, *Dermatophagoides farinae*, or both,^9^ which are the 2 most important HDM species.^10^ Moreover, it has been reported that high IgE antibody titers to HDM allergen extracts were common and increased the risk for acute wheezing among asthmatic children.^11^

Since 1980, when the first allergen was described for *D pteronyssinus*,^12^ the molecular structures of several HDM allergens have been identified, including a recently described new major HDM allergen, Der p 23.^13,14^ With the availability of a comprehensive set of HDM allergens, it is now possible to study molecular reactivity profiles that might be associated with certain manifestations of HDM-induced allergic symptoms. A study using recombinant allergen molecules to investigate potential differences in adults and children with various respiratory manifestations of HDM-associated respiratory allergy indicated lower allergen-specific IgG antibody levels in children with asthma exacerbations.^15^

The Immuno Solid-phase Allergen Chip (ImmunoCAP ISAC; Thermo Fisher Scientific, Uppsala, Sweden) technology allows determination of a patient's IgE and IgG reactivity profile to a large number of natural and recombinant allergens in a single step by using only minimal amounts of serum.^16^ Comparison of the ImmunoCAP ISAC with other techniques of allergen-specific IgE determinations, such as ImmunoCAP, showed similar diagnostic performances.^17-19^ Several studies have shown the usefulness of the chip technology for detecting allergen-specific IgE,^18^ longitudinally assessing IgE reactivity profiles,^20^ guiding the prescription of specific immunotherapy,^21^ analyzing allergen-specific IgE and IgG responses in parallel,^22^ analyzing IgE reactivity in children and adults,^23^ monitoring IgE reactivities during childhood,^24^ and predicting the development of allergy in children.^25^

The currently available ImmunoCAP ISAC 112 is able to detect IgE and IgG against 112 different allergens, including the *D pteronyssinus* allergens Der p 1, Der p 2, and Der p 10. To perform a more profound analysis of sera from patients with HDM allergy, we used the ImmunoCAP ISAC technology to produce an experimental allergen chip, which contains an extended panel of HDM allergens (ie, Der p 1, Der p 2, Der p 5, Der p 7, Der p 10, Der p 21, and Der p 23). This microarray was used to measure IgE and IgG responses in 2 groups of clinically well-defined children with HDM allergy with or without HDM-induced asthma and, for control purposes, a group of nonatopic children.

## Methods

### Subjects

Sera from 105 allergic children with HDM-associated asthma were randomly picked from the Multicentre Asthma Genetics in Childhood study. This group was termed asthmatic children with HDM allergy. Sera from 53 children with HDM allergy but without asthma were randomly picked from the International Study of Asthma and Allergies in Childhood Phase II population as age- and sex-matched nonasthmatic group. The latter group was termed nonasthmatic children with HDM allergy. From the same study population, sera from 53 nonatopic children (no allergic symptoms and negative skin prick test responses) were randomly picked. The combined use of both study cohorts (Multicentre Asthma Genetics in Childhood and International Study of Asthma and Allergies in Childhood Phase II) and previous allergy testing has been described earlier.^26-28^ A description of the criteria for patient selection can be found in the Methods section in this article's Online Repository at www.jacionline.org. Table I summarizes the demographic, serologic, and clinical data of the 3 groups. Anonymized sera were sent to Vienna, where the analyses were retrospectively performed in a blinded manner with permission from the Ethics Committee of the Medical University of Vienna.

### Natural and recombinant HDM allergens

nDer p 1 was purified by means of affinity chromatography with mAb 4C1.^29^ rDer p 2 was expressed in *Escherichia coli* as a His-tagged protein and purified, as previously described.^30^ rDer p 5, rDer p 7, rDer p 10, rDer p 21, and rDer p 23 were expressed in *E coli* as nonfusion proteins and purified to homogeneity by using ion exchange chromatography, as previously described.^14,31-33^ The proteins were stored at −20°C until use.

### ImmunoCAP ISAC microarray

For the analysis of the sera from the nonasthmatic and asthmatic groups, a customized version of the ImmunoCAP ISAC microarray termed CD chip (Thermo Fisher Scientific, Uppsala, Sweden) containing a panel of *D pteronyssinus* allergens (ie, nDer p 1, rDer p 2, rDer p 5, rDer p 7, rDer p 10, rDer p 21, and rDer p 23) and several other allergens and allergen-derived peptides (see Table E1 in this article's Online Repository at www.jacionline.org) was produced. Detection of IgE and IgG binding was performed according to the manufacturer's instructions for ISAC 112, with slight modifications. Washing steps were done exclusively with washing solution (no second washing step with water), and the chips were dried by means of centrifugation (1 minute at 1000*g* and RT). Levels of allergen-specific IgE and IgG antibodies were reported in ISAC standardized units (ISU) with a cutoff of 0.3 ISU. The allergen-specific IgE and IgG levels of the sera from the 53 nonatopic children were tested with the MeDALL allergen chip, which equals the CD chip in technical aspects but comprises additional allergens.^34^

### Basophil activation tests

Rat basophil leukemia cells (RBL-2H3)^35^ transfected with human FcεRI were sensitized with sera from asthmatic children with HDM allergy (dilution 1:10). Cell degranulation was induced by adding 3 different concentrations (ie, 10, 1, and 0.1 ng/mL of each allergen) of 2 HDM allergen mixes (mix 1: nDer p 1 and rDer p 2; mix 2: nDer p 1, rDer p 2, rDer p 5, rDer p 7, rDer p 21, and rDer p 23) or buffer as a control. β-Hexosaminidase release was measured, as previously described.^36^

### Statistics

IBM SPSS Statistics 20 (SPSS, Chicago, Ill) was used for evaluating the following statistical parameters. Differences in IgE- and IgG-binding frequencies of asthmatic, nonasthmatic, and nonatopic children with HDM allergy were assessed by using multiple χ^2^ testing or the Fisher exact test, depending on the number of observations. Differences in IgE and IgG levels among the 3 groups were analyzed with the Mann-Whitney *U* test. The correlation between IgE and IgG reactivity to the allergens was evaluated by calculating the Spearman rank correlation coefficient (ρ). A *P* value of less than .05 was considered statistically significant.

## Results

### Asthmatic children with HDM allergy more frequently have an IgE response to each of the HDM allergens and recognize more allergens than nonasthmatic children with HDM allergy

**Fig E1:**
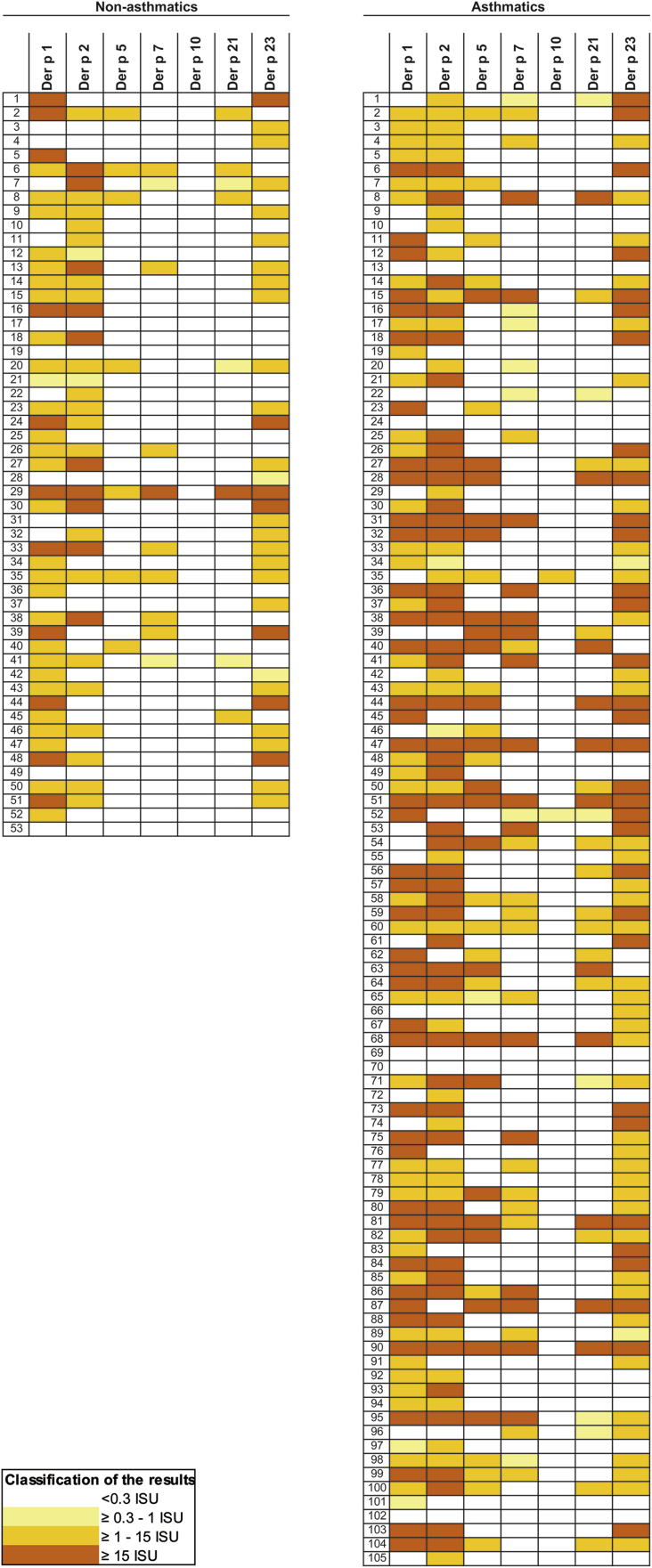


**Fig 1:**
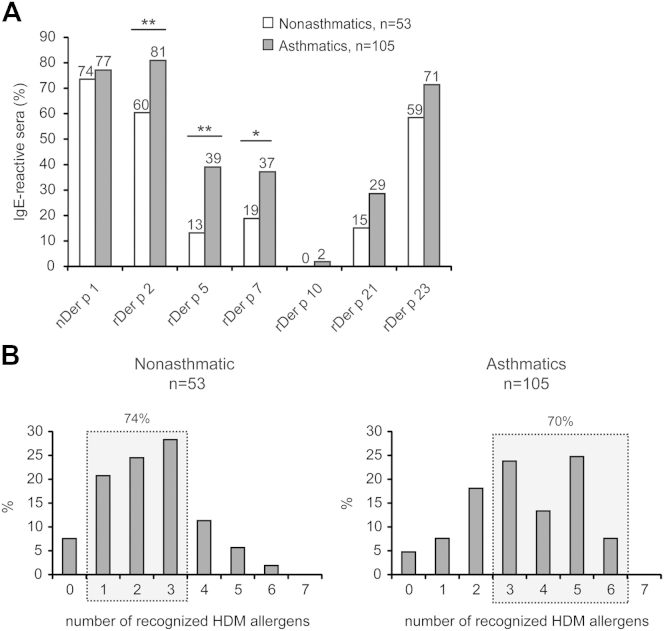
IgE reactivity of asthmatic and nonasthmatic children with HDM allergy to individual HDM allergens. **A,** IgE binding prevalences (*y-axis*: percentage) to the allergens as measured with ImmunoCAP ISAC technology for nonasthmatic *(white bars)* and asthmatic *(gray bars)* children with HDM allergy. Significant differences between the 2 groups are shown. *P < .05 and **P < .01. **B,** Number of HDM allergens *(x-axes)* recognized by nonasthmatic *(left panel)* or asthmatic *(right panel)* children with HDM allergy (*y-axes*: percentages of IgE-reactive patients). *Gray boxes* denote numbers of allergens recognized by 74% and 70% of the nonasthmatic and asthmatic children, respectively.

We found that 149 (94.3%) of the 158 analyzed children with HDM allergy had IgE reactivity to at least one of the 7 spotted HDM allergens (nDer p 1, rDer p 2, rDer p 5, rDer p 7, rDer p 10, rDer p 21, and rDer p 23). With a combination of nDer p 1 and rDer p 2, 89% of the patients were diagnosed (see Fig E1 in this article's Online Repository at www.jacionline.org). Sera from 8 children with negative chip results could be tested by means of IgE immunoblotting to *D pteronyssinus* allergen extract, and 7 showed very weak IgE reactivity (data not shown). Twenty-one percent of the patients were exclusively sensitized to nDer p 1, rDer p 2, or both. Interestingly, 18 children with HDM allergy reacted neither with nDer p 1 nor with rDer p 2. Six of these patients showed an exclusive sensitization to rDer p 23 but not to the other tested HDM allergens (see Fig E1). With a combination of nDer p 1, rDer p 2, and rDer p 23, 92.4% of the patients were identified, and only 2 patients showed exclusive sensitization to other tested HDM allergens (see Fig E1). nDer p 1 was the most frequently recognized allergen (nonasthmatic patients, 74%; asthmatic patients, 77%), followed by rDer p 2 (60% and 81%, respectively) and rDer p 23 (59% and 71%, respectively; Fig 1, *A*). rDer p 5 (nonasthmatic patients, 13%; asthmatic patients, 39%), rDer p 7 (19% and 37%, respectively), and rDer p 21 (15% and 29%, respectively) were less frequently recognized, and only a few patients had IgE reactivity to rDer p 10 (0% and 2% respectively; Fig 1, *A*). In the nonatopic group only 2 children had weak IgE reactivity, one to nDer p 1 (0.37 ISU) and one to rDer p 2 (2.11 ISU), whereas the remaining 51 had no IgE reactivity to any of the 7 tested HDM allergens (data not shown).

The frequencies of IgE recognition of the individual HDM allergens differed considerably between children with HDM allergy with and without asthma. rDer p 2, rDer p 5, and rDer p 7 were significantly more often recognized by asthmatic patients (rDer p 2, *P* = .005; rDer p 5, *P* = .001; and rDer p 7, *P* = .019; Fig 1, *A*). Interestingly, rDer p 5 was 3-fold more often recognized by asthmatic than nonasthmatic children.

Accordingly, we found that the sum of IgE reactivities to HDM components was significantly higher in the asthmatic than in the nonasthmatic group (asthmatic mean, 67.9 ISU; nonasthmatic mean, 26.7 ISU; *P* < .001), which was also reflected by a significant difference in total IgE levels between the groups (asthmatic mean, 786 kU/L; nonasthmatic mean, 338 kU/L; *P* < .001; Table I). Neither in the asthmatic group nor in the nonasthmatic group were statistically significant sex-related differences found regarding the frequency of IgE recognition of the tested HDM allergens or allergen-specific IgE levels (data not shown). There was also no correlation between age and the number of recognized allergens (all children, ρ = 0.137, *P* = .086; nonasthmatic children, ρ = 0.007, *P* = .961; asthmatic children, ρ = 0.119, *P* = .228). Finally, we checked whether different sensitization profiles in the groups could be due to differences regarding time points of blood sampling in the seasons of the year, but there were no significant differences (multiple χ^2^ testing: spring, *P* = .136; summer, *P* = .061; autumn, *P* = .462; winter, *P* = .432), especially not for autumn, where HDM burden is highest in middle Europe.^37,38^

Calculation of the odds ratio (OR) showed that patients with IgE reactivity to rDer p 5 have a more than 4-fold higher risk of asthma than patients without IgE reactivity to rDer p 5 (OR, 4.21; 95% CI, 1.74-10.22; *P* = .0015). Furthermore, the risk of being asthmatic was more than 2-fold higher in patients with IgE reactivity to rDer p 2 (OR, 2.79; 95% CI, 1.34-5.82; *P* = .006) and patients with IgE reactivity to rDer p 7 (OR, 2.54; 95% CI, 1.15-5.62; *P* = .021), respectively. The calculated ORs for the other HDM allergens (nDer p 1, rDer p 10, rDer p 21, and rDer p 23) were statistically not significant.

The positive predictive value, providing information about the probability that a positive test result correctly indicates that a certain symptom (ie, asthma) is present, was calculated for the individual allergens. Patients with IgE reactivity to rDer p 5 had a probability of 85% of having asthma. In addition, high positive predictive values were found for rDer p 7 (80%) and rDer p 21 (79%). However, the sensitivities for each of the allergens were low: rDer p 5, 39%; rDer p 7, 37%; and rDer p 21, 29%. The sensitivity could be increased to 57% when the latter 3 allergens were combined.

Another interesting finding was that asthmatic children with HDM allergy had IgE reactivity to more HDM allergens than nonasthmatic children (Fig 1, *B*). Seventy percent of the children in the asthma group reacted to 3 to 6 of the tested allergens, whereas approximately the same percentage (74%) of nonasthmatic children had IgE reactivity only to 1 to 3 HDM allergens (Fig 1, *B*, gray boxes). No IgE reactivity was found to several of the unrelated food allergens and food allergen–derived peptides that were part of the array in any of the tested patients ensuring specificity (data not shown).

### Asthmatic children with HDM allergy have higher IgE levels to HDM allergens than children with HDM allergy but without asthma

**Fig 2:**
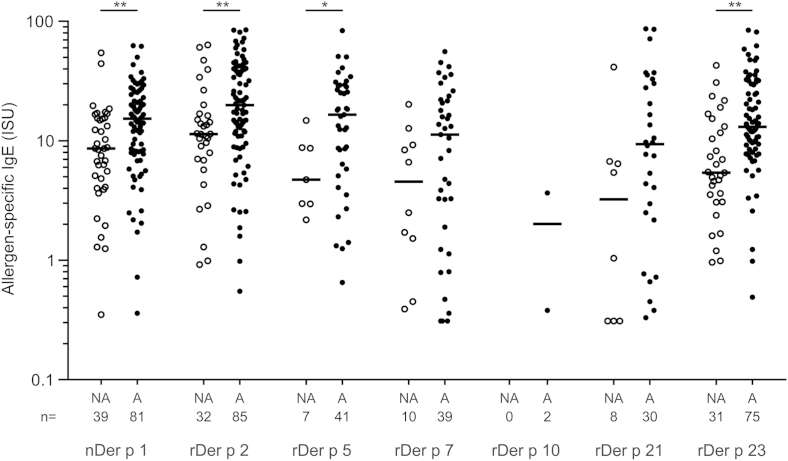
IgE levels to individual HDM allergens. Specific IgE levels (in ISU, *y-axis*) to HDM allergens *(x-axis)* determined for reactive children with HDM allergy with (*A*, *right columns*) and without (*NA*, *left columns*) asthma are displayed as scatter plots. The number of IgE-positive sera for the individual allergens are displayed below. Statistically significant differences between the 2 groups are indicated. *P < .05 and **P < .01.

Levels of allergen-specific IgE antibodies in children with HDM allergy were highest for rDer p 2 (medians: all, 15.1 ISU; asthmatic children, 19.9 ISU; nonasthmatic children, 11.4 ISU), followed by rDer p 5 (medians: all, 13.1 ISU; asthmatic children, 16.5 ISU; nonasthmatic children, 4.7 ISU) and nDer p 1 (medians: all, 13.1 ISU; asthmatic children, 15.3 ISU; nonasthmatic children, 8.6 ISU; Fig 2). Striking differences were found between HDM-allergic children with and without asthma. Asthmatic children with HDM allergy had higher IgE levels to each of the tested HDM allergens than nonasthmatic children. In particular, IgE levels to nDer p 1 (*P* = .002), rDer p 2 (*P* = .007), rDer p 5 (*P* = .031), and rDer p 23 (*P* < .001) were significantly higher in children with HDM allergy with asthma than in those without (Fig 2).

### An allergen mix comprising all respiratory HDM allergens induces stronger basophil activation than a mix containing only Der p 1 and Der p 2

**Fig E2:**
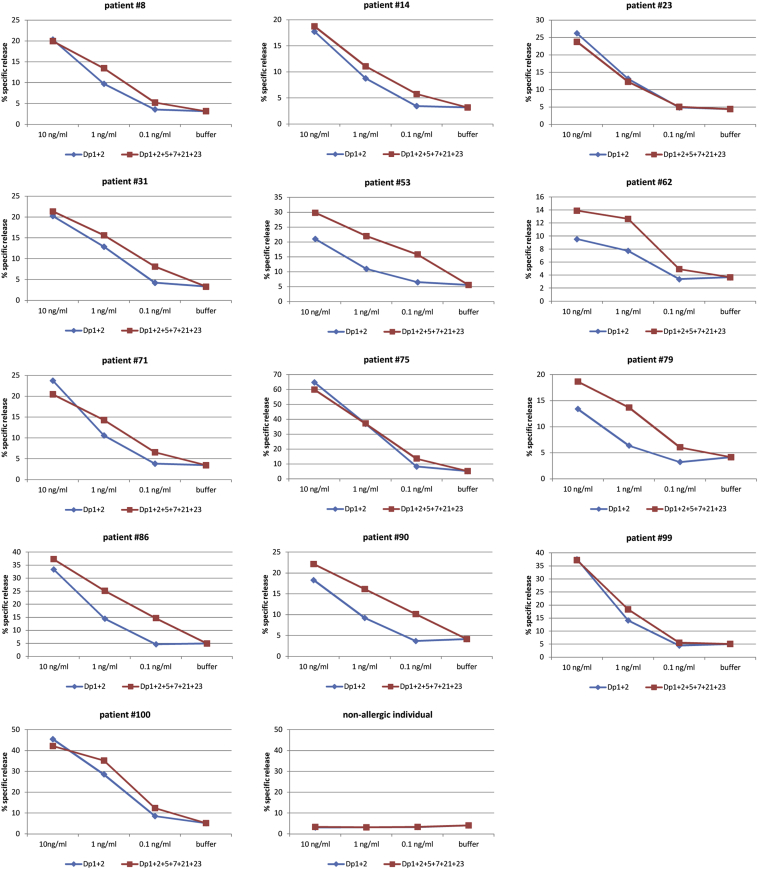


Basophil activation tests were performed to study whether sensitization to a larger number of HDM allergens and increased HDM-specific IgE levels might also have functional relevance. RBL cells expressing the human FcεRI receptor were loaded with serum IgE from 13 asthmatic children from whom serum was available and who were sensitized against a broad spectrum of HDM allergens. Then cells were exposed either to a mix containing only nDer p 1 and rDer p 2 or to a mix containing Der p 1, 2, 5, 7, 21, and 23. At a concentration of 1 ng/mL (ie, increasing part of the dose response curve), the mix containing all allergens induced a significantly higher release of β-hexosaminidase (mean relative increased release, 46%) than the mix containing only nDer p 1 and rDer p 2 in 11 of the 13 patients (see Fig E2 in this article's Online Repository at www.jacionline.org).

### Fewer asthmatic children with HDM allergy have IgG reactivity to HDM allergens than nonasthmatic children with HDM allergy, but allergen-specific IgG levels were comparable

**Fig E3:**
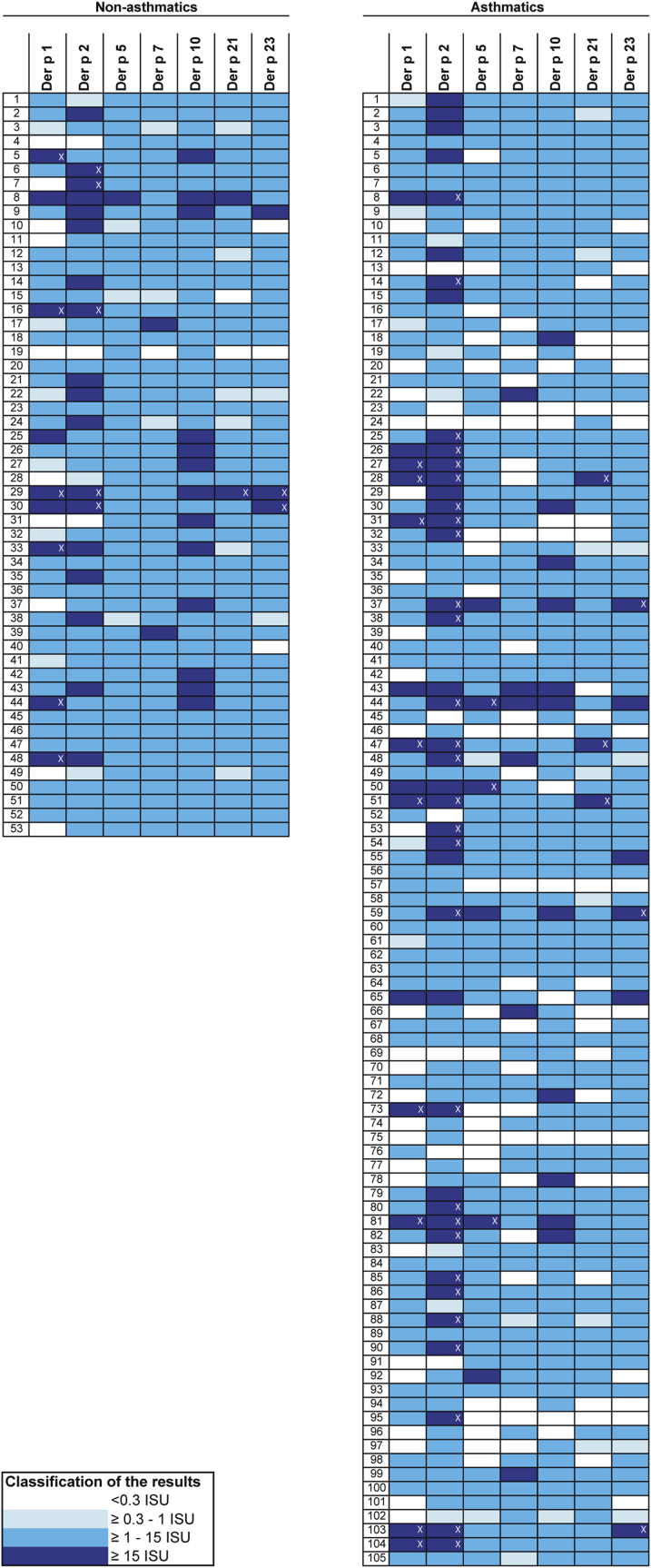


**Fig 3:**
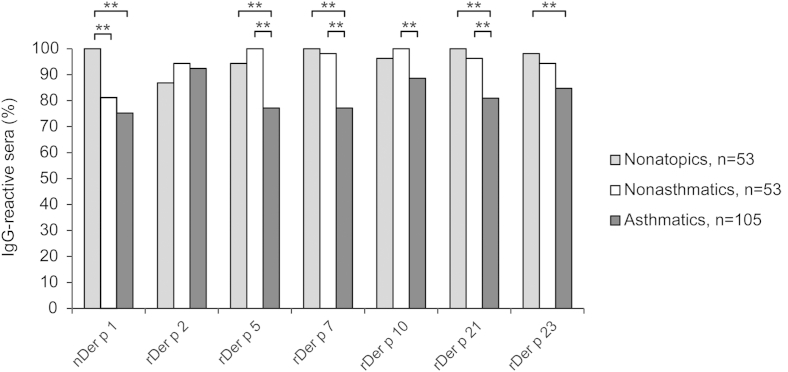
Frequencies of IgG reactivities to individual HDM allergens in asthmatic and nonasthmatic children with HDM allergy, as well as nonatopic subjects. IgG-binding frequencies (*y-axis*: percentages) to the allergens *(x-axis)* are shown for nonatopic *(light gray)*, nonasthmatic *(white bars)*, and asthmatic *(dark gray bars)* patients with HDM allergy. Statistically significant differences between the groups are indicated. **P < .01.

The IgG recognition frequency of HDM allergens was very high in patients with HDM allergy, as well as in nonatopic subjects (Fig 3 and see Fig E3 in this article's Online Repository at www.jacionline.org). The highest IgG-binding frequencies (100%) were found for rDer p 5 and rDer p 10 in the nonasthmatic group, as well as for nDer p 1, rDer p 7, and rDer p 21 in the nonatopic children. Interestingly, no patient from the nonasthmatic group had shown IgE reactivity to rDer p 10.

Although the IgG-binding frequencies were comparable between nonasthmatic and nonatopic children, the group of asthmatic children with HDM allergy had lower IgG-binding frequencies to most of the HDM allergens compared with the other 2 groups (nDer p 1: 75% vs 81% and 100%; rDer p 2: 92% vs 94% and 87%; rDer p 5: 77% vs 100% and 94%; rDer p 7: 77% vs 98% and 100%; rDer p 10: 89% vs 100% and 96%; rDer p 21: 81% vs 96% and 100%; and rDer p 23: 85% vs 94% and 98%). Statistically significant differences among the 3 groups are indicated in Fig 3.

**Fig 4:**
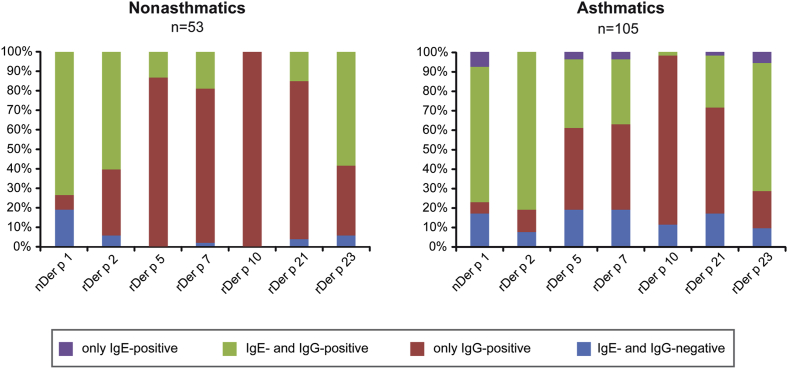
Percentages of sera with certain IgE and IgG reactivity profiles to HDM allergens. Displayed are percentages *(y-axes)* of sera among the group of asthmatic and nonasthmatic children with HDM allergy containing only IgE *(violet)*, IgE and IgG *(green)*, only IgG *(red)* or neither IgE nor IgG *(blue)* to each of the allergens *(x-axes)*.

We then analyzed the percentages of sera containing IgE and IgG antibodies, only IgG, only IgE or neither IgE nor IgG antibodies specific for each of the HDM allergens (Fig 4). There were more only IgG-positive sera in the nonasthmatic group, whereas only IgE-positive sera were found only in the asthmatic group, which also contained more sera that were IgE and IgG negative to each of the HDM allergens except Der p 1 (Fig 4).

**Fig 5:**
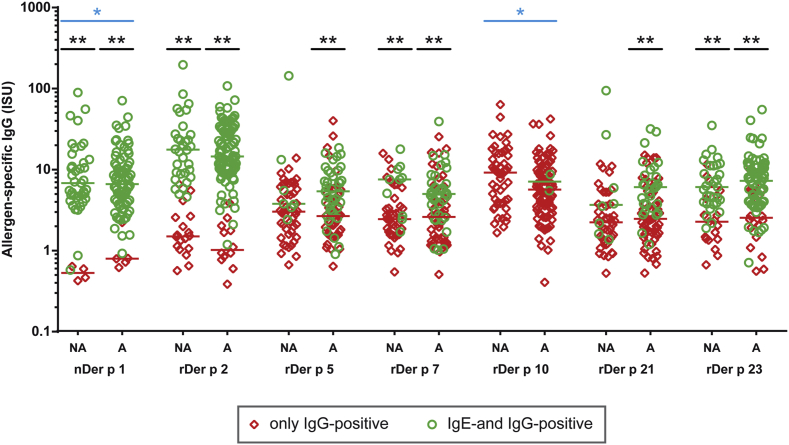
Comparison of allergen-specific IgG levels in nonasthmatic and asthmatic children with HDM allergy containing only allergen-specific IgG or allergen-specific IgE and IgG. Specific IgG levels *(y-axes)* are displayed for each of the HDM allergens *(x-axes)* for the nonasthmatic (*NA*, *left columns*) and asthmatic (*A*, *right columns*) groups. Medians are indicated, and significant differences between only IgG-positive *(red diamonds)* and IgE- and IgG-positive *(green circles)* sera are indicated by *asterisks*. **P < .01. Significant differences between nonasthmatic and asthmatic children in the IgG levels in the only IgG-positive groups (for Der p 1 and Der p 10) are indicated *(blue asterisks)*. *P < .05.

When patients were grouped into those who only had allergen-specific IgG without specific IgE or those with allergen-specific IgE and IgG antibodies, we noted that for most allergens, allergen-specific IgG levels were significantly higher in the latter group (Fig 5). Although fewer asthmatic children had IgG reactivity to the HDM allergens compared with the nonasthmatic children (Fig 3), there were no relevant differences regarding IgG levels specific for each of the tested allergens, regardless of whether patients with only positive IgG results or patients with both positive IgE and positive IgG results were analyzed. Only for nDer p 1 and rDer p 10 were the IgG levels in the only IgG-positive groups different between asthmatic and nonasthmatic children (nDer p 1, *P* = .019; rDer p 10, *P* = .014; Fig 5).

**Fig E4:**
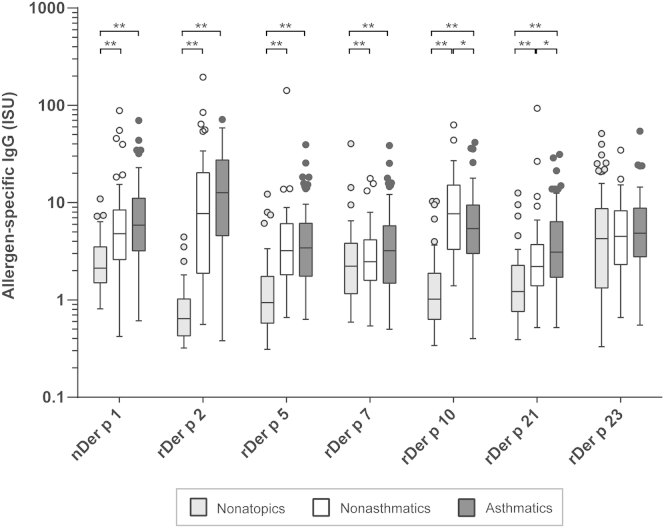


Significantly lower HDM allergen-specific IgG levels were found in the nonatopic children compared with the asthmatic and nonasthmatic groups with HDM allergy for all allergens except rDer p 23 (see Fig E4 in this article's Online Repository at www.jacionline.org).

### Allergen-specific IgE and IgG reactivities are only partially correlated

**Fig 6:**
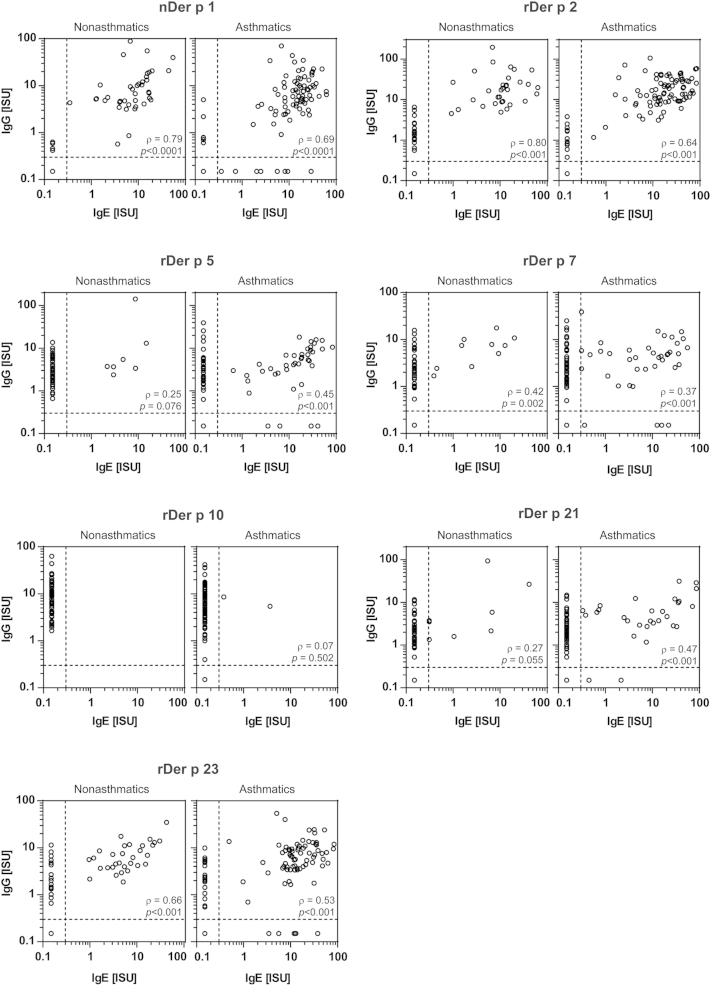
Correlations between IgE *(x-axes)* and IgG *(y-axes)* antibody levels to individual allergens for nonasthmatic *(left panels)* and asthmatic *(right panels)* children with HDM allergy are displayed as scatter plots. IgE- and/or IgG-negative values are depicted as 0.15 ISU (half of the cutoff value) to allow a log-scale presentation of the data. The correlation coefficient ρ and levels of significance (*P* values) are shown in the *lower right corner* of the graphs.

Next, we assessed correlations between IgE and IgG reactivities and levels in sera from the children with HDM allergy to the 7 tested HDM allergens (Fig 6 and see Figs E1 and E3). Figs E1 and E3 show that several asthmatic children lacked allergen-specific IgG but mounted allergen-specific IgE responses (nDer p 1: patients 75, 77, 78, 83, 91, 92, 97, and 101; rDer p 5: patients 32, 46, 95, and 98; rDer p 7: patients 17, 40, 75, and 95; rDer p 21: patients 64 and 95; rDer p 23: patients 18, 57, 66, 75, 78, and 95). The partial dissociation of allergen-specific IgE and IgG responses became also apparent when allergen-specific IgE and IgG levels were correlated (Fig 6). In the nonasthmatic group significant correlations between allergen-specific IgE and IgG levels were found only for rDer p 2 (ρ = 0.80, *P* < .001) and nDer p 1 (ρ = 0.79, *P* < .0001). The correlations were less pronounced for rDer p 23 (ρ = 0.66, *P* < .001) and rDer p 7 (ρ = 0.42, *P* = .002) and only very weak for rDer p 21 (ρ = 0.27, *P* = .055) and rDer p 5 (ρ = 0.25, *P* = .076). None of the patients with HDM allergy without asthma had IgE reactivity to rDer p 10, but all had IgG reactivity (Fig 6). Notably, rDer p 10–specific IgG levels were very high. Only rDer p 2–specific IgG levels were higher than rDer p 10–specific IgG levels (Fig 5).

In agreement with the fact that fewer asthmatic patients had allergen-specific IgG responses and higher levels of allergen-specific IgE in the asthma group, the correlation between allergen-specific IgE and IgG levels was weaker for the asthmatic group than for the group of nonasthmatic subjects for most of the tested allergens (nDer p 1: ρ = 0.69, *P* < .0001; rDer p 2: ρ = 0.64, *P* < .001; rDer p 7: ρ = 0.37, *P* < .001; rDer p 10: ρ = 0.07, *P* = .502; and rDer p 23: ρ = 0.53, *P* < .001). Only for rDer p 5 (ρ = 0.45, *P* < .001) and rDer p 21 (ρ = 0.47, *P* < .001) was the correlation higher in the asthma group (Fig 6). However, several children from the asthmatic and nonasthmatic groups mounted high allergen-specific IgE levels, as well as high IgG levels (see Fig E3, marked by x).

## Discussion

In this study we used a panel of 7 microarrayed HDM allergens to study possible differences in IgE and IgG recognition in clinically well-defined children with HDM allergy with or without allergic asthma. Our study revealed interesting differences regarding IgE recognition in these 2 groups. Asthmatic children with HDM allergy showed a more broadly spread recognition of different allergens, as well as higher allergen-specific IgE levels, compared with the nonasthmatic group.

Both high IgE levels and broad recognition of HDM allergens should lead to dense loading of mast cells, basophils, and also antigen-presenting cells with allergen-specific IgE through their IgE receptors and render such patients more sensitive to allergen exposure. In fact, earlier studies performed by measuring HDM-specific IgE levels with allergen extracts also showed that high levels of HDM-specific IgE antibodies are associated with reduced lung function and increased risk of wheezing.^11,39^ However, these studies were performed with allergen extracts and therefore did not provide information regarding numbers of recognized allergens. The possibility of analyzing IgE reactivities to a large panel of different allergens with small amounts of serum is a strength of the ImmunoCAP ISAC chip technology. ImmunoCAP ISAC technology also offers high sensitivity, specificity, and good performance regarding intra-assay and interassay variation.^34^ However, it must be borne in mind that results depend on the use of high-quality allergen preparations, the coupling must be optimized for each of the components, and because of the low amount of allergen on the solid phase, IgG might compete with IgE binding, leading to lower IgE signals.^34^

One novel aspect of our study is the demonstration that asthmatic children compared with nonasthmatic children were more often sensitized against several allergens, among them the major allergen rDer p 2 and rDer p 5, rDer p 7, and rDer p 21, which were shown to be potent allergens regarding the elicitation of immediate-type allergic reactions.^32,40^ The lower prevalence of IgE reactivity to rDer p 2 in our study compared with another study^41^ might be explained by geographic differences, as has already been shown by Weghofer et al.^42^

The finding that a mix comprising Der p 1, 2, 5, 7, 21, and 23 induced stronger basophil activation in polysensitized patients than a mix comprising only Der p 1 and Der p 2 suggests that polysensitization might eventually have functional consequences, but this needs to be investigated in clinical studies.

The fact that the IgE response of the asthmatic children was more broadly spread toward several different allergen molecules compared with that of the nonasthmatic children did not seem to be due to different immunogenicity of the individual allergens in the 2 patient groups. On the contrary, we found an inverse situation for allergen-specific IgG recognition in the 2 groups. Nonasthmatic children even more often had IgG reactivity against the individual allergens compared with asthmatic children. Therefore there are several mutually nonexclusive possibilities concerning why asthmatic children have IgE reactivity to more HDM allergens and produce higher allergen-specific IgE levels compared with nonasthmatic children. One possibility is that certain HDM allergens, such as the cysteine protease Der p 1, could act as a kind of “initiator allergen” by disrupting tight junctions, thus reducing epithelial barrier function and leading to increased intrusion of sensitizing allergens.^43^ It is also possible that certain HDM allergens have intrinsic properties rendering them capable of pushing the innate immune system toward a T_H_2 pathway.^44,45^ Furthermore, allergic patients who are predisposed to asthma might exhibit intrinsic features, such as epithelial barrier defects, increased atopic predisposition, or both, that render them more susceptible to allergic sensitization.^46,47^ The net result of these mechanisms would be a more frequent and more intense IgE recognition of individual allergens present in a complex allergen source, such as HDM, which is preferentially associated with asthma, whereas sensitization to fewer allergens is more related to rhinitis. It has been suggested that children might acquire additional IgE sensitizations with increasing age,^24^ and it is therefore possible that the fact that asthmatic children reacted with more HDM allergens could be due to a bias toward higher age in this group. However, when we correlated the children's age with the number of recognized allergens, we found no correlation between age and the number of recognized allergens. In addition to age, we studied also whether differences regarding blood sampling in the seasons might have had an effect on IgE recognition profiles in the asthmatic and nonasthmatic groups, but no significant differences regarding blood sampling in the 4 seasons were found in the groups.

Because the asthmatic children had significantly higher HDM-specific IgE levels, it was not surprising to find that they exhibited also higher total IgE levels compared with the nonasthmatic group because it is known that most of the total IgE consists of allergen-specific IgE.^48,49^

Regarding the naturally occurring allergen-specific IgG responses, we noted that for each of the tested allergens, with the exception of Der p 10, allergen-specific IgG levels were higher in patients who also produced allergen-specific IgE against the same allergen. This observation might be explained by different routes of sensitization because Der p 1, Der p 2, Der p 5, Der p 7, Der p 21, and Der p 23 are mainly present in fecal particles and hence could sensitize preferentially through the respiratory tract, yielding stronger and more frequent IgE responses. By contrast, Der p 10 is mainly present in mite bodies and hence can sensitize through the skin or gut by involving cross-reactivity with homologous food allergens.^50^ Thus Der p 10–specific IgG responses might also result from contact with cross-reactive food allergens, such as tropomyosins from seafood. For most of the HDM allergens, the frequency of IgG reactivity was comparable between the nonatopic and atopic nonasthmatic children, but IgG levels were lowest for the nonatopic children. A limitation of our study is that no data regarding allergen exposure were available, and therefore we can only speculate about whether lower exposure, lower immunogenicity, or both of the HDM allergens might have caused differences in HDM allergen–specific IgE and IgG responses.

In summary, this study has revealed that the assessment of IgE and IgG reactivities toward a comprehensive panel of HDM allergen components defines complex serologic reactivity profiles that might help to discriminate asthmatic and nonasthmatic children. It will now be interesting to perform longitudinal studies in birth cohorts to answer the question of whether such multiallergen tests might be able to predict the development and eventually persistence of asthma.^51,52^

**Clinical implications**

IgE sensitization to a broad panel of HDM allergens and high IgE levels to certain components are associated with allergy affecting the lower airways in children with HDM allergy.

## Figures and Tables

**Table I:** Demographic, serologic, and clinical characterization of the study population

	Nonatopic subjects			Nonasthmatic subjects			Asthmatic subjects		
Male	Female	Total	Male	Female	Total	Male	Female	Total
Patients, no. (%)	25 (47)	28 (53)	53 (100)	25 (47)	28 (53)	53 (100)	50 (47)	55 (53)	105 (100)
Age range (y [minimum-maximum])	9-11	9-11	9-11	9-12	9-11	9-12	7-17	7-16	7-17
Mean age (y)	9.68	9.61	9.64	9.76	9.71	9.74	10.68	10.36	10.5
Mean total IgE (kU/L)	78.77	163.72	121.24	363.8	315.1	338.5	659.5	901.9	786.4
>1 Hospital visit in last 12 mo due to asthma, no. (%)	0 (0)	0 (0)	0 (0)	0 (0)	0 (0)	0 (0)	22 (44.0)	21 (38.2)	43 (41.0)
>3 Hospital visits in last 12 mo due to asthma, no. (%)	0 (0)	0 (0)	0 (0)	0 (0)	0 (0)	0 (0)	9 (18.0)	8 (15.5)	17 (16.2)
Atopic dermatitis, no. (%)	0 (0)	0 (0)	0 (0)	4 (16.7)	6 (22.2)	10 (18.9)	27 (56.3)	27 (50)	54 (51.4)
Sensitization to other respiratory allergens									
Cat, no. (%)	0 (0)	0 (0)	0 (0)	13 (52.0)	15 (53.6)	28 (53)	28 (56.0)	38 (69.1)	66 (63)
Grass pollen, no. (%)	0 (0)	0 (0)	0 (0)	18 (72.0)	20 (71.4)	38 (72)	36 (72.0)	41 (74.5)	77 (73)
Tree pollen, no. (%)	0 (0)	0 (0)	0 (0)	8 (32.0)	13 (46.2)	21 (40)	30 (60.0)	30 (54.5)	60 (57)

## Supplementary data

Online Repository Data
