# Supplementary material for: Different IgE recognition of mite allergen components in asthmatic and nonasthmatic children
Source: J Allergy Clin Immunol. 2015 Oct;136(4):1083–91. doi: 10.1016/j.jaci.2015.03.024 (PMC4595482; doi:10.1016/j.jaci.2015.03.024)
Supplement: Online Repository Data [file mmc1.docx]

**E1. METHODS**

Subjects

Subjects investigated in the present study were selected from the Multicenter Asthma Genetic in Childhood Study (MAGICS) and the German leg of the International Study of Asthma and Allergies in Childhood phase II (ISAAC II) as previously described^E1-4^ and summarized^E5^:

In MAGICS, 1255 children were recruited between 2001 and 2007 from seven different asthma clinical centers located in Germany or Austria. Asthma was diagnosed in 865 children (mean age 11 years) by pediatric pulmonologists based on physical examination, clinical history, lung function tests, and allergy status. In ISAAC II, a large cross-sectional study conducted in 1995 and 1996, 5629 school children aged between 9–11 years, were recruited in Munich and Dresden (Germany).^E3^ The methodology of the ISAAC II study has been described in details elsewhere.^E3, 4^ In this study, asthma status was assessed by the parents of the children, who reported a physician’s diagnosis of asthma once or recurrent spastic or asthmatic bronchitis more than once. Allergic rhinitis was defined by the presence of doctor’s diagnostic report in the parental questionnaire.

Allergic sensitization against common inhalant allergens, *D. pteronyssinus*, *D. farinae*, cat epithelium, grass pollen and birch pollen, was tested in MAGICS by measuring specific serum IgE using CLA-1 (Hitachi Chemical Diagnostics, UK) or AllergyScreen (Mediwiss Analytic, Germany) panels. In ISAAC II, atopic sensitization was assessed by skin prick testing against common allergens, such as *D. pteronyssinus*, *D. farinae*, *Alternaria tenuis*, cat dander, mixed grass and mixed tree pollen, and/or by Sx1 assay (ImmunoCAP, Phadia, Germany) containing a cocktail of antibodies against allergens, including local grass pollen, rye pollen, birch pollen, mugwort pollen, *D. pteronyssinus*, cat dander, dog dander and *Cladosporium herbarum*.

Atopic asthma was equal to the presence of both asthma and atopy, the latter defined by the positive results for specific IgE against at least one of allergens tested (RAST/CAP class equivalent of >2) in MAGICS.

**TABLES**

**Table E1.** List of allergens spotted on the CD chip

| **Allergen source** | **Allergen/protein** | **Rec./ natural or synthetic** | **Function of the protein** | **Reference** |
| --- | --- | --- | --- | --- |
| Wheat | clone #10 | R | Serine proteinase inhibitor | Constantin *et al.*, Allergy, 2009 |
|  | clone #37 | R | Thioredoxin H |  |
|  | clone #38 | R | Glutathione transferase |  |
|  | clone #112 | R | 1-Cys-peroxiredoxine |  |
|  | clone #123 | R | Profilin |  |
|  | clone #126 | R | Dehydrin |  |
|  | Tri a 37 | R | α-purothionin | Pahr *et al.*, J Allergy Clin Immunol , 2013 |
|  | Tri a 36 (191-369) | R | Fragment of LMW glutenin | Baar *et al.*, J Immunol, 2012 |
|  | Tri a 36 | R | LMW glutenin | Baar *et al.*, J Immunol, 2012 |
| Bee | Api m 1 | N | Phospholipase A2 | Mittermann *et al.*, J Allergy Clin Immunol, 2010 |
|  | Api m 1 | R | Phospholipase A2 (prod. in *E. coli*) |  |
|  | Api m 2 | R | Hyaluronidase (prod. in insect cells) |  |
|  | rApi m 2 | R | Hyaluronidase (prod. in *E. coli*) |  |
| Pineapple | Bromelain | N | cysteine endopeptidase from pineapple stem |  |
| Wasp | Ves v 1 | R | Phospholipase A1 | Valenta *et al.*, unpublished |
|  | Ves v 5 | R | Antigen 5 | Mittermann *et al.*, J Allergy Clin Immunol, 2010 |
| House dust mite | Der p 1 | N | Cysteine protease | Hales *et al.*, Clin Ex Allergy, 2000 |
|  | Der p 2 | R | ML domain lipid binding protein | Chen *et al.*, Mol Immunol, 2008 |
|  | Der p 5 | R | Unknown | Weghofer *et al.*, Int Arch Allergy Immunol, 2008 |
|  | Der p 7 | R | Unknown | Resch *et al.*, Clin Exp Allergy, 2011 |
|  | Der p 10 | R | Tropomyosin | Resch *et al.*, Clin Exp Allergy, 2011 |
|  | Der p 21 | R | Unknown | Weghofer *et al.*, Allergy, 2008 |
|  | Der p 23 | R | Chitin-binding protein | Weghofer et al., J Immunol, 2013 |
| Timothy grass | Phl p 4 | N | Berberine bridge enzyme | Stumvoll *et al.*, Biol Chem, 2002 |
|  | Phl p 13 | N | Polygalacturonases | Swoboda *et al.*, J Immunol, 2004 |
|  | Phl p 13 | R | Polygalacturonases |  |
| Ragweed | Amb a 1 | R | Pectate lyase | Biomay AG (Vienna, Austria) |
| Dog | Can f 2 | R | Lipocalin | Valenta *et al.*, unpublished |
| Cat | Fel d 2 | N | Serum Albumin | Reininger *et al.*, Clin Exp Allergy, 2003 |
|  | Fel d 2 | R | Serum Albumin |  |
| Carp | Cyp c 1 | R | Parvalbumin | Swoboda *et al.*, J Immunol, 2002 |
| Human | Hom s 2 | R | NAC-alpha Protein | Mittermann *et al.*, J Investig Dermatol, 2008 |
| *S. aureus* | Fibronectin-binding protein | R | Fibronectin-binding protein | Reginald *et al.*, J Allergy Clin Immunology, 2011 |
| Cow's milk | Bos d 4 | N | α-lactalbumin | Sigma Aldrich (St Louis, MO, USA) |
|  | Bos d 5 | N | β-lactoglobulin, variant A |  |
|  | Bos d 5 | N | β-lactoglobulin, variant B |  |
|  | Bos d 6 | N | bovine serum albumin |  |
|  | Bos d 8 | N | caseins (α-, β-, κ-caseins) |  |
|  | Bos d lactoferrin | N | Transferrin |  |
|  | Bos d 4 | R | α-lactalbumin | Hochwallner *et al.*, Clin Exp Allergy, 2010 |
|  | Bos d 5 | R | β-lactoglobulin |  |
|  | Bos d 9 | R | αS1-casein |  |
|  | Bos d 10 | R | αS2-casein |  |
|  | Bos d 11 | R | β-casein |  |
|  | Bos d 12 | R | κ-casein |  |
| Milk peptides | Bos d 9-derived peptide 1 | S |  | Schulmeister *et al.*, J Immunol, 2009 |
|  | Bos d 9-derived peptide 1.2 | S |  |  |
|  | Bos d 9-derived peptide 2 | S |  |  |
|  | Bos d 9-derived peptide 2.3 | S |  |  |
|  | Bos d 9-derived peptide 3 | S |  |  |
|  | Bos d 9-derived peptide 3.4 | S |  |  |
|  | Bos d 9-derived peptide 4 | S |  |  |
|  | Bos d 9-derived peptide 4.5 | S |  |  |
|  | Bos d 9-derived peptide 5 | S |  |  |
|  | Bos d 9-derived peptide 5.6 | S |  |  |
|  | Bos d 9-derived peptide 6 | S |  |  |
|  | Bos d 5-derived peptide 1 | S |  | Valenta *et al.*, unpublished |
|  | Bos d 5-derived peptide 2 | S |  |  |
|  | Bos d 5-derived peptide 3 | S |  |  |
|  | Bos d 5-derived peptide 4 | S |  |  |
|  | Bos d 5-derived peptide 5 | S |  |  |
|  | Bos d 5-derived peptide 5a | S |  |  |
|  | Bos d 5-derived peptide 6 | S |  |  |
|  | Bos d 5-derived peptide 7 | S |  |  |
|  | Bos d 5-derived peptide 8 | S |  |  |
|  | Bos d 5-derived peptide 8a | S |  |  |
|  | Bos d 5-derived peptide 9 | S |  |  |
|  | Bos d 5-derived peptide 10 | S |  |  |
|  | Bos d 5-derived peptide 11 | S |  |  |
|  | Bos d 4-derived peptide 1 | S |  | Hochwallner et al., J Allergy Clin Immunol, 2010 |
|  | Bos d 4-derived peptide 2 | S |  |  |
|  | Bos d 4-derived peptide 3 | S |  |  |
|  | Bos d 4-derived peptide 4 | S |  |  |
|  | Bos d 4-derived peptide 5 | S |  |  |
|  | Bos d 4-derived peptide 6 | S |  |  |
|  | Bos d 4-derived peptide 7 | S |  |  |
|  | Bos d 4-derived peptide 8 | S |  |  |

**REFERENCES**

E1. Moffatt MF, Kabesch M, Liang L, Dixon AL, Strachan D, Heath S, et al. Genetic variants regulating ORMDL3 expression contribute to the risk of childhood asthma. Nature 2007; 448:470-3.

E2. Michel S, Liang L, Depner M, Klopp N, Ruether A, Kumar A, et al. Unifying candidate gene and GWAS Approaches in Asthma. PLoS One 2010; 5:e13894.

E3. Asher MI, Keil U, Anderson HR, Beasley R, Crane J, Martinez F, et al. International Study of Asthma and Allergies in Childhood (ISAAC): rationale and methods. Eur Respir J 1995; 8:483-91.

E4. Weiland SK, Bjorksten B, Brunekreef B, Cookson WO, von Mutius E, Strachan DP. Phase II of the International Study of Asthma and Allergies in Childhood (ISAAC II): rationale and methods. Eur Respir J 2004; 24:406-12.

E5. Sharma V, Michel S, Gaertner V, Franke A, Vogelberg C, von Berg A, et al. A role of FCER1A and FCER2 polymorphisms in IgE regulation. Allergy 2014; 69:231-6.

FIGURE LEGENDS

FIG E1. Heat map representation of the IgE reactivities of non-asthmatic and asthmatic HDM-allergic children to individual allergens. Patients´ IgE levels (Non-asthmatics: 1-53; Asthmatics: 1-105) to the allergens are indicated (colour code).

**FIG E2.** Basophil degranulation experiments. Mediator release induced by three different concentrations (*x-axes*) of HDM allergens (mix 1: Der p 1 and Der p 2; mix 2: Der p 1, Der p 2, Der p 5, Der p 7, Der p 21 and Der p 23) are displayed as percentages of total β-hexosaminidase release *(y-axes:* means of triplicates*)* for asthmatic children and for a non-allergic individual.

FIG E3. Heat map representation of the IgG reactivities of non-asthmatic and asthmatic HDM-allergic children to individual allergens. Patients´ IgG levels (Non-asthmatics: 1-53; Asthmatics: 1-105) to the allergens are indicated (colour code). Sera containing IgG and IgE levels =/> 15 ISU specific for certain allergens are indicated by “x”.

**FIG E4.** IgG levels to individual HDM allergens. Specific IgG levels (ISU, *y*-axis) to HDM allergens (*x*-axis) determined for non-atopic children (light grey bars, left column), non-asthmatic children (white bars, middle column) and asthmatic children (black bars, right column) are displayed as box plots containing 50% of the data. Outliers greater than 1.5 times of the interquartile range (IQR) are identified with circles. Statistically significant differences between the two groups are indicated (**p* < 0.05, ***p* < 0.01).
